# Emotional Influences on Cognitive Flexibility Depend on Individual Differences: A Combined Micro-Phenomenological and Psychophysiological Study

**DOI:** 10.3389/fpsyg.2019.01138

**Published:** 2019-05-24

**Authors:** Alejandra Vásquez-Rosati, Rodrigo Montefusco-Siegmund, Vladimir López, Diego Cosmelli

**Affiliations:** ^1^Escuela de Psicología, Pontificia Universidad Católica de Chile, Santiago, Chile; ^2^Laboratorio de Fenomenología Corporal, Santiago, Chile; ^3^Escuela de Kinesiología, Facultad de Medicina, Universidad Austral de Chile, Valdivia, Chile; ^4^Centro Interdisciplinario de Neurociencias, Pontificia Universidad Católica de Chile, Santiago, Chile; ^5^Instituto Milenio para la Investigación en Depresión y Personalidad, MIDAP, Santiago, Chile

**Keywords:** cognitive flexibility, emotions, music, micro-phenomenological interview, P300, neurophenomenology

## Abstract

Imagine a scenario where you are cooking and suddenly, the contents of the pot start to come out, and the oven bell rings. You would have to stop what you are doing and start responding to the changing demands, switching between different objects, operations and mental sets. This ability is known as cognitive flexibility. Now, add to this scenario a strong emotional atmosphere that invades you as you spontaneously recall a difficult situation you had that morning. How would you behave? Recent studies suggest that emotional states do modulate cognitive flexibility, but these findings are still controversial. Moreover, there is a lack of evidence regarding the underlying brain processes. The purpose of the present study was, therefore, to examine such interaction while monitoring changes in ongoing cortical activity using EEG. In order to answer this question, we used two musical stimuli to induce emotional states (positive/high arousal/open stance and negative/high arousal/closed stance). Twenty-nine participants performed two blocks of the Madrid Card Sorting Task in a neutral silence condition and then four blocks while listening to the counterbalanced musical stimuli. To explore this interaction, we used a combination of first-person (micro-phenomenological interview) and third-person (behavior and EEG) approaches. Our results show that compared to the positive stimuli and silence condition, negative stimuli decrease reaction times (RTs) for the shift signal. Our data show that the valance of the first emotional block is determinant in the RTs of the subsequent blocks. Additionally, the analysis of the micro-phenomenological interview and the integration of first- and third-person data show that the emotional disposition generated by the music could facilitate task performance for some participants or hamper it for others, independently of its emotional valence. When the emotional disposition hampered task execution, RTs were slower, and the P300 potential showed a reduced amplitude compared to the facilitated condition. These findings show that the interaction between emotion and cognitive flexibility is more complex than previously thought and points to a new way of understanding the underlying mechanisms by incorporating an in-depth analysis of individual subjective experience.

## Introduction

To successfully navigate everyday life, we must continuously balance the need to fulfill a myriad of internal motivations with the capacity to react to a changing environment. As we confront the world, we choose many of the things we do, think or act upon, but we must also react to unexpected changes, adapting our behavior and, not infrequently, changing plans. In many such situations, adaptive behavior is, from a cognitive perspective, the result of being able to inhibit an ongoing action and effectively reorient our resources to deal with whatever the novel situation turns out to be (Diamond, 2013).

The skill that allows us to change between stimuli, operations, and mental sets is known as cognitive flexibility (Lin et al., 2013). It is usually studied through the use of task-switching paradigms: participants must adopt a certain mental set in order to accomplish a given task, but then must change it in response to a cue or an instruction, in order to continue to succeed. Most tasks used for studying cognitive flexibility are variations of the classical sorting card task (Berg, 1948; Grant and Berg, 1948; Kiesel et al., 2010; Doebel and Zelazo, 2015). Here, participants must sort a sequence of cards according to one of many possible characteristics (color, for example). Mid-way through the task, and upon being instructed, participants must start sorting the cards according to a different rule (e.g., number). Trials in which the rule must be changed (i.e., switch trials) take longer to solve than those in which the rule must be repeated, a quantity known as switch cost. Errors too are more likely in switch trials than in repetition trials.

Card sorting tasks have been extensively studied from behavioral and neuropsychological testing perspectives (Dehaene and Changeux, 1991; Barceló, 2003; Doebel and Zelazo, 2015; see Nyhus and Barceló, 2009 for an overview). From the point of view of underlying brain activity (Barceló and Rubia, 1998; Barceló et al., 2000; Barceló, 2003; Adrover-Roig and Barceló, 2010; Gajewski and Falkenstein, 2017), switch trials are accompanied by an event-related potential around 300 ms after the onset of a cue (also known as P3), a positivity that is absent in repetition trials. Some authors have reported this P3 as being the result of two different components, a frontally distributed potential that appears at 300–400 ms and is called P3a and a latter and posteriorly distributed potential called P3b. Traditionally, P3a activity has been related to novelty (Schroger and Wolff, 1998; Friedman et al., 2001), whereas P3b to context updating (Donchin and Coles, 1988, 1998). However, not all studies have named this potential consistently, sometimes referring to it as a “cue-locked positivity” (Karayanidis et al., 2010) or a switch-related positivity (Rushworth et al., 2002, 2005; Miniussi et al., 2005; Li et al., 2012).

Yet, in daily life, our choices, our changes of mind and the many ways we deal with the different challenges we face, do not occur in a vacuum. We encounter the world as living, sentient beings and as such, we do so from a point of view that always carries with it an emotional tone and a bodily disposition (Cosmelli and Thompson, 2010). This embodied encounter is not an added ingredient to a cognitive agent, but a condition of possibility of any meaningful interaction in the world (Damasio, 1994; Colombetti and Thompson, 2008; Colombetti, 2010). In other words, cognition and emotion are deeply intertwined and shape together our behavior, a fact that has received substantial empirical support from several branches of psychology and cognitive and affective neuroscience (Pessoa, 2008; Storbeck and Clore, 2008; Todd and Anderson, 2011; Todd et al., 2012a). It is therefore all the more surprising that how emotions affect cognitive flexibility, such a core skill for adapted behavior, has received little attention.

Despite the existence of a number of studies dealing with the effects of emotional arousal on a diversity of cognitive processes ranging from perception to decision making (Mather and Sutherland, 2011; Todd et al., 2012b), there are only a few studies that deal with the specific interaction between cognitive flexibility and emotions. Demanet et al. (2011) showed that the level of arousal of an affective stimulus can produce a change in switch costs, while the valence dimension seems to affect global task performance. On the contrary, Lin et al. (2013) showed that positive affect promotes cognitive flexibility. In addition, there is evidence that points to the effects of permanent emotional moods, such as anxiety, on cognitive flexibility. Compared to high levels of anxiety, low levels of anxiety seem to promote cognitive flexibility (Ansari et al., 2008; Derakshan et al., 2009). It is unclear where these differences are coming from. Among other possibilities, it could be that the presentation of an emotional stimulus is insufficient to generate a true internal change (or a transient emotional state) that can affect task-switching performance. Moreover, to our knowledge, there is still no evidence showing how specific emotional states could modulate the neural dynamics of task switching processes.

In this study, we were interested in elucidating if and how emotional states can affect cognitive flexibility as measured by a task switching paradigm, and how this would be observed in terms of the underlying brain activity. Taking into account the contradictory results that have been reported, we also aimed at developing a more in-depth approach to the emotional experience, based on a systematic investigation into the first-person experience that participants had during the task. We did this because evoking a specific emotional response can be highly dependent on the individual, the inducing stimulus and/or the experimental conditions (Gabrielsson, 2002; Lutz et al., 2002). As mentioned above, it could be that some of the inconsistencies in previous studies stem from the fact that not all participants experience emotional inductions in the same way. Also, participants could be merely recognizing emotions but not experiencing them as such, which would amount to a different type of intervention.

To address these questions, we used a version of the card sorting task that has been adapted for psychophysiological recordings (Barceló, 2003), and paired it with an emotional induction based on musical stimuli. Usually, musical stimuli used to induce emotions are fragments of highly stereotyped popular songs that, by virtue of them being widely known, can easily be related to personal episodes that bias emotional states (see for example the music used in Stephens et al., 2010). To avoid this effect on the emotional induction (i.e., how I should feel *versus* how I actually feel), the stimuli used in this study were novel for all participants. All musical stimuli were designed and validated to induce two specific emotional dispositions: positive valence, high arousal, and open stance or negative valence, high arousal and closed stance (Vásquez-Rosati, 2017). The “stance” dimension refers to the postural/bodily disposition that is associated with a given emotion: while an open stance is associated with the feeling of an overall extension of the body and an availability to stimulation, a closed stance is associated with a feeling of inward retraction of the limbs and protection from stimulation (Kim and Andre, 2008). To assess these dimensions, we used a modified version of the Self Assessment Mannequin scale (SAM; Bradley and Lang, 1994; Vásquez-Rosati, 2017). In addition to the standard arousal and valence dimensions, the modified SAM scale evaluates the bodily disposition evoked by the musical stimulus to assess the open/closed stance dimension.

In order to ensure a consistent emotional experience throughout the entire block, we presented acoustic and visual stimulation during an emotional induction phase before the task (de Gelder and Vroomen, 2000; Baumgartner et al., 2006; Spreckelmeyer et al., 2006; Logeswaran and Bhattacharya, 2009). Then, to aid in sustaining of the emotional disposition without interfering, only the musical stimulus was maintained while the participant undertook the task. Finally, we used a micro-phenomenological interview (Petitmengin and Bitbol, 2009; Petitmengin and Lachaux, 2013) to gather in-depth experiential, first-person information about the emotional experience and its relation with task performance. As we hope to demonstrate, this approach can provide a much richer account of how emotional disposition can affect cognitive flexibility, complementing and extending the traditional psychophysiological approach.

## Materials and Methods

### Participants

Twenty-six participants (12 females; mean age 28.3 ± 5.2 years, range 20–41 years) took part in the study. They had normal or corrected-to-normal visual acuity and no history of neurological or psychiatric disorder. Three of them had musical and instrument studies and two of them had only instrument studies. Twenty-one participants reported listening to music while doing other tasks. All participants enjoyed listening to music. The study reviewed and approved by the Ethics Committee of the Escuela de Psicología, from the Pontificia Universidad Católica de Chile. All participants read and signed an informed consent form before their participation in the actual experiment.

### Stimuli

#### Musical Stimuli

In a previous study, in collaboration with a musician and composer, we designed two musical stimuli to induce opposite emotional states: negative valence, high arousal, closed stance (stimulus A); and positive valence, high arousal, open stance (stimulus B). These were validated through self-report questionnaires and first-person descriptions (Vásquez-Rosati, 2017). The “white” noise used in this experiment was the same used by Stephens et al. (2010) and Nyklicek et al. (1997) (70 dB intensity). Both music and white noise clips were presented with headphones from a 16-bit and 44,100 Hz stereo file during the entire block. The sound of the music was kept between 50–90 dB, but adjusted subjectively for each participant before the beginning of the experimental session, in order to ensure a comfortable and constant level throughout the experiment.

#### Affective Images

We selected 92 images from the International Affective Picture System (IAPS; Lang et al., 2008), 46 affective images of positive valence and high arousal (mean valence = 7.19 ± 0.55; mean arousal = 5.94 ± 0.75) and 46 affective images of negative valence and high arousal (mean valence = 2.53 ± 0.07; mean arousal = 6.20 ± 0.65). Each set of affective images was separated into two groups of 23 images, maintaining the same level of affective valence and arousal between them, according to the database score. The images were presented twice each, for a duration ranging from 3 to 5 s randomly. Therefore, each induction totaled approximately 3 min.

#### Madrid Card Sorting Task (MCST)

We used the MCST developed by Barceló (2003), which is a computer version of the Wisconsin Card Sorting Test (WCST) adapted to the study of concurrent Event-Related Potentials (ERPs). The MCST consists of 24 cards from the original set of WCST. Each card can be unequivocally matched with one of the four key cards according to one of the dimensions of the stimulus (color, shape or number). These 24 cards are used repeatedly in the 137 pseudo-randomized trials, in 18-series arrays. The classification rule is unknown to the subject and changes randomly from one series to the next. In each series array, the participant has two trials to find the new rule (shif1, shift2). Once the new rule is identified, a variable number of repetition trials must be completed (rep1, rep2, rep3, rep4 – last). Participants sat one meter away from the computer screen and stimuli were adjusted in size to reproduce the viewing conditions of the original experiment (4° horizontally and 3.5° vertically; Barceló, 2003). Presentation Neurobehavioral System^^®^^ software was used to present the entire task. The task was applied identically to the original except for feedbacks: auditory feedbacks were replaced by visuals feedbacks (“+” stay feedback, “o” shift feedback y “x” error feedback) in order not to interfere with the ongoing music. Participants responded using both hands and a four-button keypad.

### Procedure and Measurements

#### Procedure

Once in the laboratory, participants signed the informed consent form and completed a personal information questionnaire to obtain demographic data and whether or not the participant had any formal musical studies. Once the EEG electrodes were installed, the participant sat in a comfortable chair in front of the screen where the task would be presented and listened to the instructions of the experiment. First, a short practice block of the task was presented to ensure that the participant had understood the instructions. The practice block was followed by two silent blocks (without music: MCST_S). Following the blocks without music, the blocks with music were presented interleaved and counterbalanced among participants (ABAB or BABA, whereby blocks are named according to the musical clip presented throughout it). Blocks with music started with a white noise, which had a variable duration between 50 and 60 s. During this stage, participants were instructed to clear their mind of thoughts, emotions, sensations, etc. Then, to induce the specific emotional state before the task-switching block itself the music and the presentation of IAPS images began. During this stage, that lasted approximately 3 min; participants were instructed to listen to the music while freely exploring the images, submerging and recognizing the sensations that appeared throughout their presentation. After the sequence of images was completed, participants pressed a button to start the actual task while the music continued in the background. After completing each task-switching block (12 min max), participants were asked to evaluate through the SAM self-report questionnaire how they felt during the execution of the task. At the end of the experiment, electrodes were removed and the micro-phenomenological interview was carried out. Finally, participants received a debriefing of the experiment.

#### EEG Measurements

EEG was recorded using the BioSemi^^®^^ ActiveTwo amplifier system from 32 scalp sites (10–20 system) with Ag/AgCl electrodes mounted on an elastic cap (Klem et al., 1999; Stern et al., 2001). Eight additional electrodes were attached to left and right mastoids, the two outer canthi of both eyes (HEOG), and infraorbital and supraorbital regions of the eye (VEOG), while a Lead II configuration on the chest was used to measure the EKG. All signals were digitalized with a sampling rate of 1024 Hz and 24 bit A/D conversion. Off-line, EEG and EOG were processed as follow: first, the data was segmented in 3 s epochs (1 s before and 2 s after card presentation) then each epoch was re-referenced. Trials that presented blinks or between 300 ms before and 600 ms after the presentation of the stimulus were eliminated. To identify and remove ocular artifacts we used an Independent Component Analysis (ICA) (Jung et al., 2000a,b). On average, we removed 1.46 (±0.81) components out of a total of 32. The resulting signals were then bandpass-filtered between 0.1–25 Hz (phase shift-free Butterworth filter; 24 dB/octave slope). After baseline correction (−500 to 0 ms), the average ERP of the artifact-free trials was calculated for each subject at each recording site. A grand average across subjects was then obtained for the two conditions of interest: shift1 (change rule, novelty) and rep2 (maintain rule, repetition). We use rep2 instead of rep1 trials because when the participant has to use a second attempt to find the rule (shift2), that shift trial will most likely be correct and thus somewhat ambiguous regarding novelty vs repetition. Accordingly, the following repetition (rep2) is the first time that the participant unambiguously repeats the rule.

#### Micro-Phenomenological Interview

The micro-phenomenological interview is a method that allows the description of a singular experience in detail, and has its historical roots in the *explicitation interview* (Vermersch, 1999, 2004; Petitmengin, 2014). The particularity of this approach is that, through open questions, it focuses on actions or processes involved in the lived experience from an embodied perspective, in order to reveal implicit aspects of it. Then, the analytical approach works across two axes: the diachronic (the temporal development of experience) and synchronic (the configuration of the experiential space) dimensions of experience (Vermersch, 1994; Petitmengin, 2006). This approach has been used to gather descriptions of emotional and bodily experience (Valenzuela-Moguillansky, 2013; Depraz and Archives, 2017; Vásquez-Rosati, 2017), seizure anticipation in epilepsy patients and its correlates with neural activity (Petitmengin et al., 2007) and meditative experience (Ataria, 2015; Petitmengin, 2017; Przyrembel and Singer, 2018).

The micro-phenomenological interview involves two main steps: the interview itself and its analysis. In the first, the interviewer guides the interviewee to the evocation of a specific situation, and from this state, he helps the description of this particular experience. During this process, the interviewer must have special care not to induce any kind of specific response. To this end, the interviewer uses “content-empty” or “structure-driven” questioning (Petitmengin and Bitbol, 2009). During the description of the experience the diachronic dimension is explored with temporal questions as “How did you start?” “What happened after/before that?” To explore the synchronic dimension, the interviewer reorients the attention of the interviewee toward the structural characteristics of a particular moment of experience using questions as “How do you see that?,” “How do you feel that?.” The interview concludes when no new information is provided by the interviewee.

The analysis is an iterative process aimed at revealing invariants in the diachronic and synchronic structures of experience. To do so, first, an individual analysis is made for each interview. To obtain the individual diachronic structure, it is necessary to transcribe the interview, select useful information (actions and processes), organize information according to the temporal development of experience and find the temporal categories or phases of experience. The individual synchronic structure can be mapped out with the information from each phase of experience or with the initially selected information. In order to find experiential categories, iterative questioning and abstraction operations are used. These results can be represented as dynamic lines or as a diagram (Valenzuela-Moguillansky and Vásquez-Rosati, 2019). Once this process is completed for all interviews, it is possible to build the generic structure of the experience. In the generic diachronic structure, individual structures are aligned according to external or internal referents (which depend on the objective of the study) to find temporal invariants. Then, the generic synchronic structure of each generic phase of experience is generated as the individual synchronic analysis (Valenzuela-Moguillansky and Vásquez-Rosati, 2019).

Results can be validated by their internal phenomenological consistency or external phenomenological consistency. In the first case, validation is obtained by the confirmation of the hypothetical structure gathered from the descriptions through the iterative questioning process. In the second case, validation is obtained by the detection of neural configurations according to phenomenological clusters (neurophenomenology) and/or inter-subjective confirmation (Petitmengin and Bitbol, 2009; Høffding and Martiny, 2015; Petitmengin et al., 2018).

In this study, 21 participants were interviewed after the experimental procedure. We were especially interested in the analysis of the synchronic dimension of the descriptions, in order to understand how the music and the subsequent emotional disposition might affect task performance. We then used this synchronic generic structure of experience to cluster and reanalyze the behavioral and electrophysiological data. As a result, we used two groups for the electrophysiological analyses (seven participants per group) and four groups for the behavioral analyses (nine, ten, two, and eight participants, respectively).

### Behavioral and Electrophysiological Data Analysis

Mean reaction times (RTs) and ERP were obtained from efficiently completed MCST series only (Barceló, 2003). RT outlier’s values were defined as those beyond two standard deviations above the average of the trial type and were removed from each participant’s data. Mean RTs were subjected to a multivariate analysis of variance (MANOVA) design with Block Order (1–6) × Condition (S, A and B) × Trial (shift1, shift2, rep1, rep2, rep3, rep4, rep5, and last) as repeated-measure factors.

From the total of twenty-six subjects, six EEG recordings were excluded from the analysis due to noisy signals, leaving a total sample of twenty subjects for the analysis. Taking into account the lack of prior results regarding the effects of emotional induction on brain activity during task-switching, and the challenges inherent to the use of first-person experiential categories to guide the neurophysiological analysis, we chose to focus on well-known ERP components. Thus, area below the curve ERP amplitudes, for feedback-locked averages, was computed in the 490–540 ms window post-stimulus onset, for the P300 component. Additionally, we performed a non-parametric cluster-based permutation test between conditions (S, A, and B) which effectively controls for the family-wise error rate (FWER; Maris and Oostenveld, 2007). All *post hoc* tests of simple effects were performed using one-way ANOVA for multiple group comparisons or *t*-test for two group comparisons.

## Results

### Emotional Disposition According to SAM Self-Report Questionnaire

**FIGURE 1:**
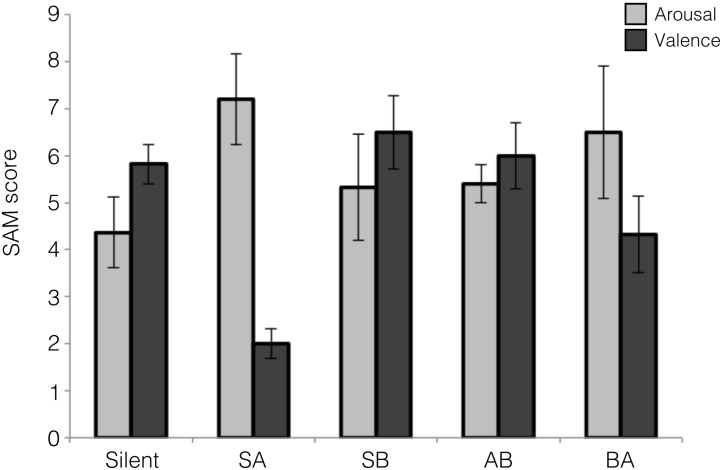
Subjective evaluation of emotional experience with SAM scale. SA: A condition preceded by silent condition, SB: B condition anteceded by silent condition, AB: B condition anteceded by A condition, and BA: A condition anteceded by B condition. The error bars correspond to the standard error of the mean.

Participants reported how they felt after each block of the experiment using a bi-dimensional scale of arousal and valence (Bradley and Lang, 1994). There were significant differences between the silent condition and the first exposition to A music (SA) in arousal (mean Silent: 4.36 (±2.5), mean SA: 7.2 (±2.2); *p* = 0.047) and valence (mean Silent: 5.8 (±1.4), mean SA: 2 (±0.7); *p* = 0.0001). In contrast, no differences were observed between the silent condition and the first exposition to B music (SB) in arousal (mean Silent: 4.36 (±2.5), mean SB: 5.3 (±3.3); *p* = 0.87) or valence (mean Silent: 5.8 (±1.4), mean SB: 6.5 (±4.1); *p* = 0.73). When comparing SA versus SB we found significant differences in valence (mean SA: 2 (±0.7), mean SB: 6.5 (±4.1) *p* = 0.004) but not in arousal (mean SA: 7.2 (±2.2), mean SB: 5.3 (±3.3); *p* = 0.13). These results show that participants felt similarly during the silent and music B conditions, while they felt less pleasure and more activation with music A, as compared to the silent condition (Figure 1). In the second emotional block, no significant differences were found between music conditions (arousal, mean AB: 5.4 (±0.9), mean BA: 6.5 (±3.7); *p* = 0.89; valence, mean AB: 6 (±1.6), mean BA: 4.3 (±2.2); *p* = 0.06).

### Behavioral Results

**FIGURE 2:**
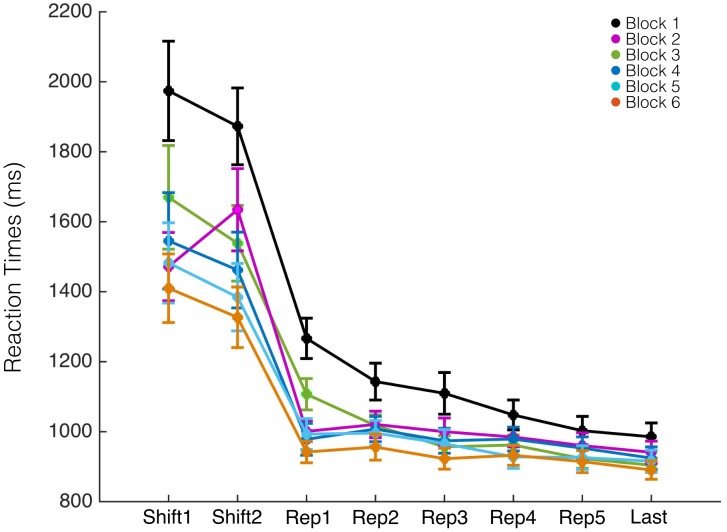
Reaction times according to experimental block presentation. The first block (in black) showed longer RTs for switch trials (shift1, shift2) and repetition trials (rep1–rep3). Error bars show the standard error of the mean.

We first compared RTs of all blocks of the task, independently of their emotional content. As expected, the RTs of all blocks were slower for shift trials and got faster throughout the repetition trials. We also found that RTs decreased as the experiment progressed, so that that the first block had slower RT in comparison to subsequent blocks (*F*_(5,149)_ = 1.59, *p* = 0.012; Figure 2). Detailed RT results for all trial types and conditions are available in the supplementary material. These differences were found for shift trials (shift1: *F*_(5,149)_ = 2.7, *p* = 0.023; shift2: *F*_(5,149)_ = 3.49, *p* = 0.0051) and for the first three repetition trials (rep1: *F*_(5,149)_ = 7.75, *p* = 0.00001; rep2: *F*_(5,149)_ = 2.59, *p* = 0.028 and rep3: *F*_(5,149)_ = 2.52, *p* = 0.032; rep4: *F*_(5,149)_ = 1.74, *p* = 0.13; rep5: *F*_(5,149)_ = 0.92, *p* = 0.47; Last: *F*_(5,149)_ = 1.17, *p* = 0.33). These results are consistent with a training effect, so that participants take more time to complete the first blocks (silent condition) than the last blocks.

**FIGURE 3:**
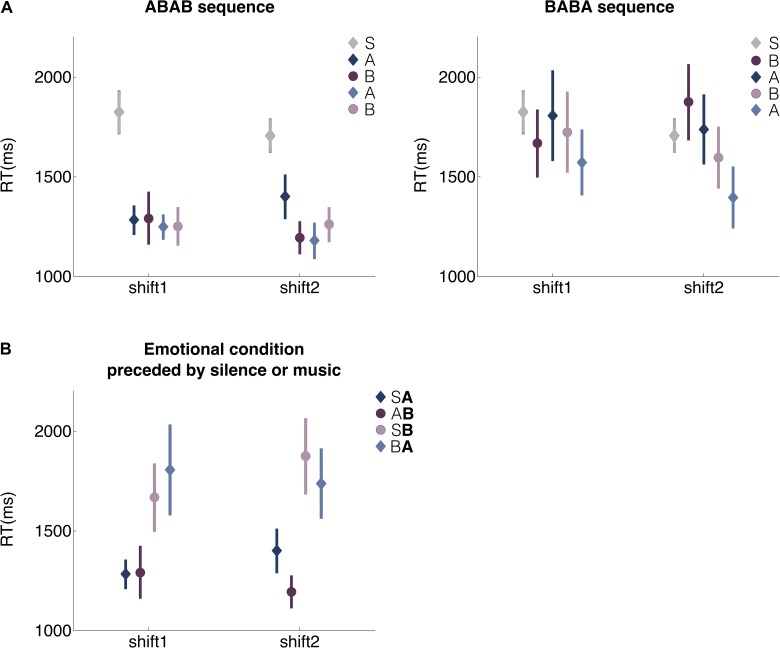
Reaction times for emotional blocks. RTs of shift trials are shown. **(A)** ABAB and BABA sequence. S: second block of the silent condition. A: block with negative music, high activation, closed stance. B: block with positive music, high activation, open stance. Shift1 and shift2: first and second rule change attempt, respectively. **(B)** RT for emotional conditions. SA: A emotional condition preceded by silence condition. AB: B emotional condition preceded by A emotional condition. SB: B emotional condition preceded by silence condition. BA: A emotional condition preceded by B emotional condition. Error bars correspond to standard error of the mean.

Regarding the effect of the emotional disposition induced during the task on performance (Figure 3A), we found an interaction effect between block order and emotional condition (*F*_(3,145)_ = 1.59, *p* = 0.039): when emotional disposition A is induced in the first block (ABAB sequence), RTs were faster compared with blocks that started with emotional disposition B (BABA sequence; *F*_(1,24)_ = 4.51, *p* = 0.042). Interestingly, this effect is not only present in the first emotional block, but can also be observed in the subsequent blocks, independent of the musical condition (shift1: *F*_(7,96)_ = 2.47, *p* = 0.022; shift2: *F*_(7,96)_ = 3.67, *p* = 0.0015). These results suggest that the first emotional disposition determines performance in successive affective blocks as if the first emotional disposition that is induced prevails over time. Furthermore, when comparing the silent (S) condition with the first induced emotional condition, RTs were faster in emotional condition A when compared to those of condition S, but no differences were found between conditions B and S (*F*_(2,49)_ = 3.89, *p* = 0.027; S vs. A *p* = 0.0096; S vs. B *p* = 0.36).

Moreover, when condition A is anticipated by condition S (SA), shift1 has faster RTs than when A is anticipated by condition B (BA; *p* = 0.0032; Figure 3B). The opposite effect is observed for shift2 trials when condition B is anticipated by condition A (AB): RTs of block B were faster than those obtained when blocks B were anticipated by condition S (SB; *p* = 0.0029). These results show that affective valence A produces faster responses as compared to conditions S and B.

### ERP Results

**FIGURE 4:**
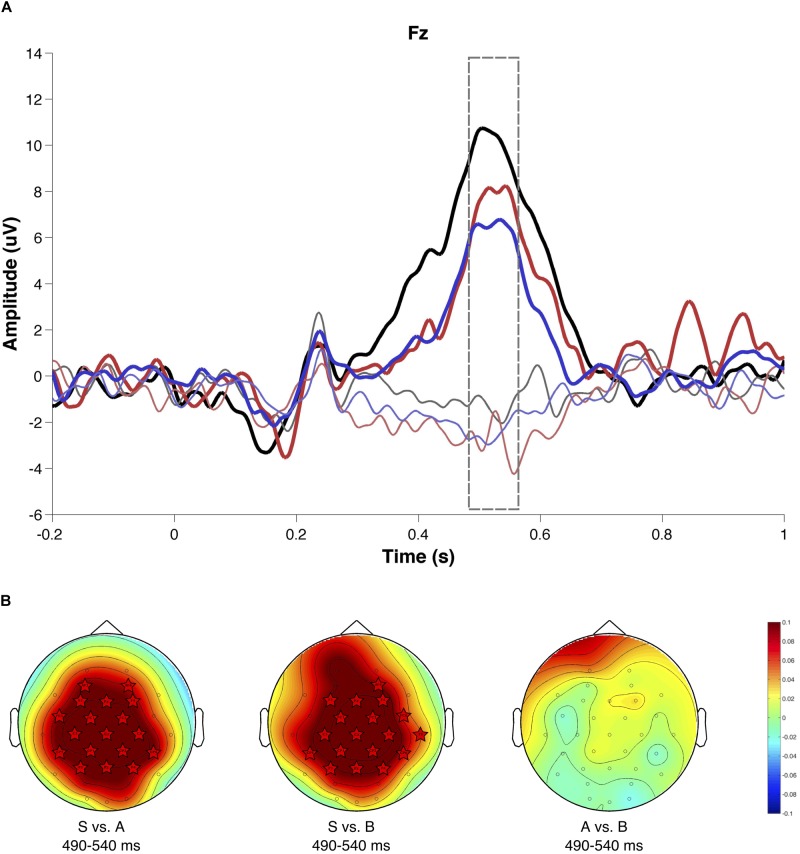
Evoked brain activity associated with shift (Shift1) and repetition (Rep2, see text for details) trials. **(A)** The temporal window for ERP analysis of P300 area under the curve is shown dashed line. Silence condition is shown in black, A condition in red, and B in blue. The darker line shows the potential associated to shift feedback, while the soft line shows the potential to the second repetition of the correct rule. **(B)** Scalp amplitude difference maps for P300 (490–540 ms): stars show electrodes that present significant differences between conditions according to a non-parametric cluster-based permutation test.

The grand-average ERP for feedback-locked events from shift1 and rep2 trials of the MCST series are shown in Figure 4A. The silent condition shows a P300 component (Fz electrode) evoked for shift1 trials (novelty) compared to repetition trials (rep2). P300 amplitude in Fz electrode is reduced in the emotional blocks, independent of their emotional valence (S vs. A *p* = 0.0097; S vs. B *p* = 0.0025; A vs. B *p* = 0.58). A cluster-based permutation test showed more sensors participating in the P300 significant cluster (*p* < 0.02), corresponding to the silent condition compared to the emotional condition, but not between emotional conditions (Figure 4B).

### Emotional Experience and Task Performance

**FIGURE 5:**
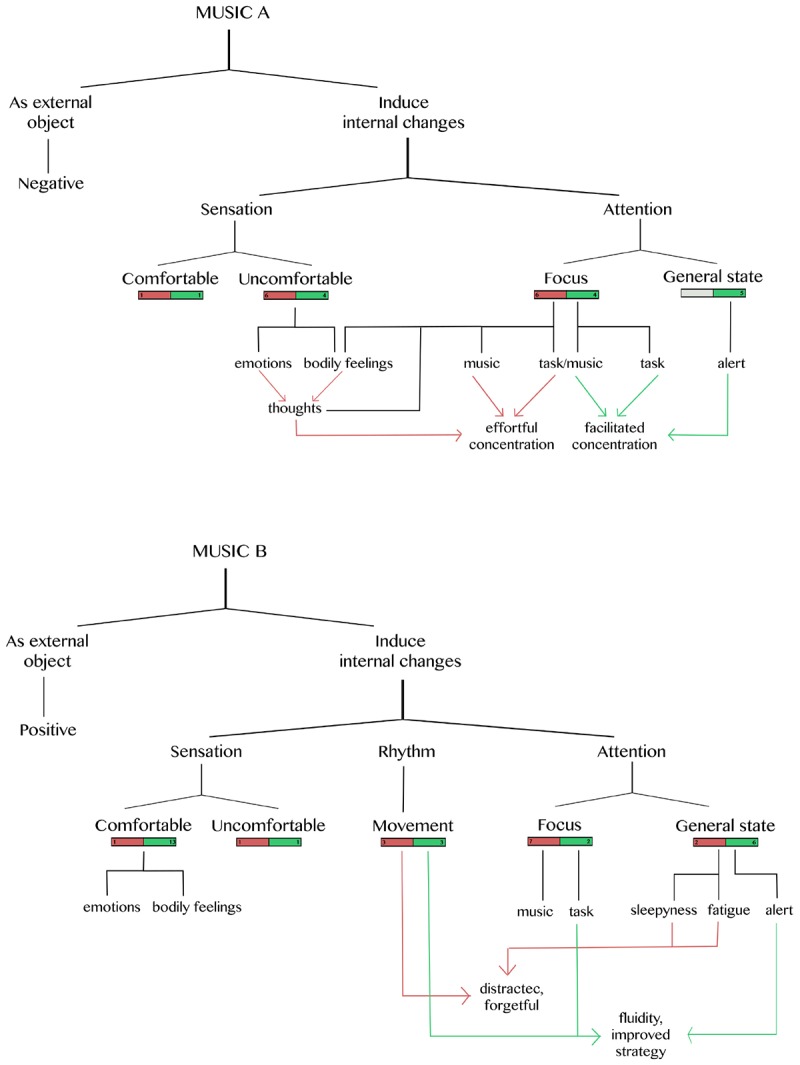
Representative scheme of the generic structure of the experience of performing the task with background music. Experiential categories that emerged from the experience are shown: squares in red show the number of participants who felt hindered during task execution; green squares show the number of participants who felt facilitated during task execution. Arrows relate the experiential categories and the level of on task concentration of the participants. **Upper panel**: music A; **Lower panel** music B.

Because we were interested in understanding how the emotional disposition induced by music might affect task performance, we considered only the synchronic information gathered through the description of the experience. The results of this analysis (Figure 5) describe in detail the experiential space generated by each music and which elements of it affect participants task performance, from their subjective point of view.

#### Music A

The first distinction that appears during the experience of doing the task with music is the difference between perceiving and feeling an emotion. In the first case, participants were capable of distinguishing emotional characteristics *in* the music (perceive), while in the second they were aware of internal changes produced *by* the music (feel). Most participants report both distinctions regarding the musical experience.

When music is perceived, a negative connotation was attributed to it. In particular, participants perceive the music as something unpleasant (*n* = 9), that gave the impression of something horrific (*n* = 5) and that stimulated aggression (*n* = 1).

On the other hand, when music-induced internal changes (*n* = 12). It was reflected specifically in sensations and in the attentional disposition. In the first case, participants described that they could feel comfortable (*n* = 2) and uncomfortable (*n* = 10) sensations. The comfortable sensations were activated and relaxed. The uncomfortable sensations were characterized by emotions such as fear (*n* = 2) and hate (*n* = 2) and/or by bodily sensations as shivering (*n* = 2), tension (*n* = 4), stress (*n* = 2) and anguish (*n* = 5). Four participants reported that they experienced this uncomfortable state as facilitating the execution of the task. This was congruent with their behavioral performance (see below). Uncomfortable bodily sensations in some cases (6) promoted negative thoughts (finish quickly, do not want to listen to music), which interfered with the execution of the task.

Regarding changes in attentional disposition, five participants described that music A led them to a general state of being alert; all of them felt that this state facilitated the execution of the task. Also, participants reported that they could have specific attentional focuses: on the task (*n* = 2, facilitated), between music and task (*n* = 6), their sensations (*n* = 2, not facilitated) and their thoughts (*n* = 2, not facilitated).

When participants felt that the emotional disposition generated by the music hindered their task performance, their reports agree in that they had to do an additional effort to concentrate on the task. To do so, they repeated internally the rule which they were selecting, while trying to disengage from the music by focusing their attention on the figures on the screen and reminding themselves to do it efficiently and quickly. Conversely, when the music facilitated the task, participants described that they were more concentrated, the rule was present to their mind or was easier to recall, and they were more easily disengaged from the music.

#### Music B

As in the description of the experience with music A, the distinction between perceiving and feeling an emotion also appears with music B. Participants perceive this music as pleasurable (*n* = 2), happy or joyful (*n* = 3) and ludic (*n* = 1). On the other hand, when music generates internal changes (*n* = 17), this was reflected in sensations (*n* = 12), attentional disposition (*n* = 6) and the appearance of an inner rhythm (*n* = 6).

Sensations were marked by a pleasant general feeling, with the appearance of emotions as happiness and joyfulness (*n* = 5) and bodily sensations of relaxation and tranquility (*n* = 9). Mostly, these pleasant sensations facilitated the execution of the task. Only two participants had uncomfortable sensations that in one case facilitated while in the other hindered the execution of the task.

On the attentional dimension, music B brought participants to an activated state of alert (*n* = 6). Participants related this state to the experience of doing the task more easily. Only one participant felt sleepy and another experienced fatigue, which in both cases were related to the experience of higher difficulty in performing the task.

The inner rhythm was described as recognizable, consonant and familiar melodies in music that generate an internal rhythm that accompanies the execution of the task, either by following the rhythm with their feet or by gently tapping the keypad. This could either facilitate task execution (*n* = 3) or act as a distracting element that made it more difficult (*n* = 3). When this emotional disposition facilitated the task (*n* = 6), participants described that the music awakened them and that they had less interior dialogue (*n* = 2); their attention was on the task, making it more fluent and improving the strategy used to resolve it (*n* = 4). On the contrary, when participants felt hampered doing the task (*n* = 9), they described that their attention was directed to the music (*n* = 7) and that they were more forgetful and distracted (*n* = 3). Participants realized that it was difficult for them to do the task because they made more mistakes (*n* = 3), forgot the rule (*n* = 1), or were not attentive to the feedback that indicated rule changes (*n* = 2).

### Behavioral and Electrophysiological Results According to Micro- Phenomenological Invariants

#### Behavioral Results

**FIGURE 6:**
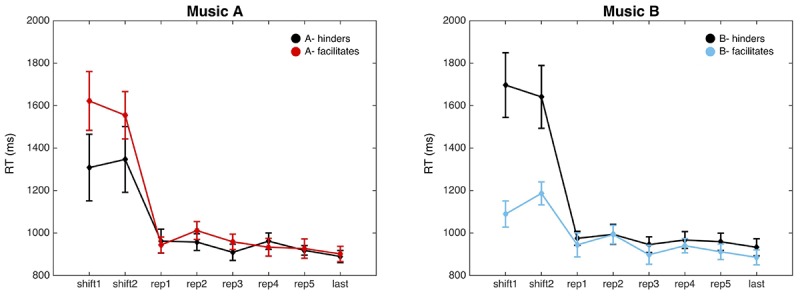
RTs analyzed based on subjective experience. The **left panel** shows the RTs when music A facilitates or hinders the execution of the task. The **right panel** shows the RTs when music B facilitates or hinders the execution of the task. The *X*-axis shows the sequence of trial types and the *Y*-axis shows the time in milliseconds that it takes for the participants to respond. Error bars present the standard error of the mean.

We classified participants according to their first-person experience in four groups: facilitated by music A (fA), facilitated by music B (fB), hindered by music A (hA) and hindered by music B (hB). As Figure 6 shows, significant differences were found between fB and hB. Shift trials show faster reaction times where participants felt facilitated by music B (shift1: *F*_(1,34)_ = 11.43, *p* = 0.0018; shift2: *F*_(1,34)_ = 6.93, *p* = 0.01265). This effect was not significant between fA and hA conditions (shift1: *F*_(1,33)_ = 2.27, *p* = 0.14; shift2: *F*_(1,33)_ = 1.23, *p* = 0.28).

#### Electrophysiological Results

**FIGURE 7:**
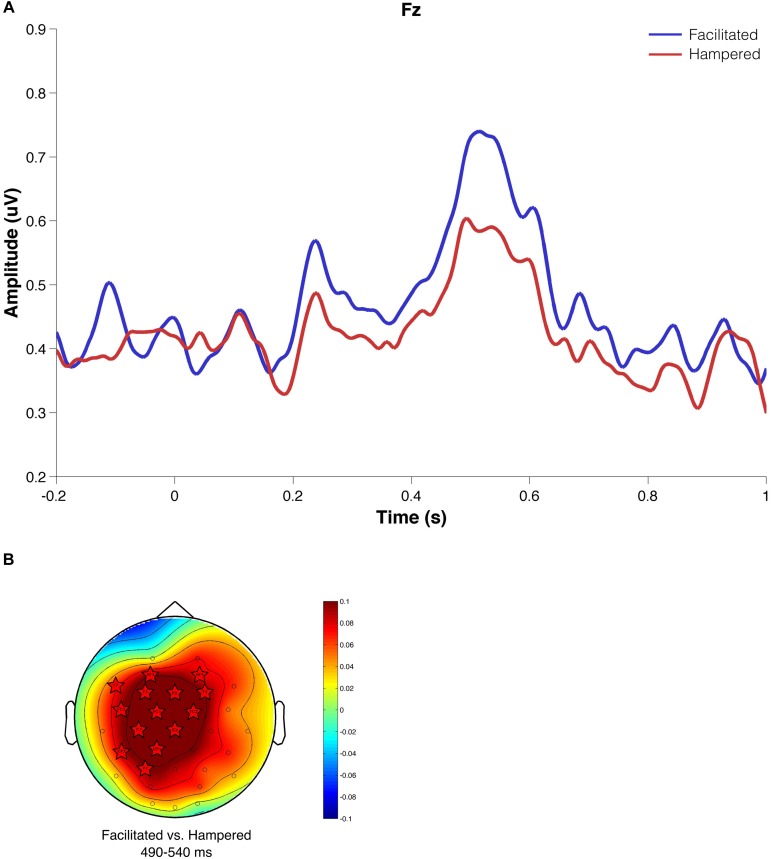
Evoked brain activity based on subjective experience. **(A)** Evoked activity to the first shift signal. The facilitated condition is shown in blue and the hampered condition in red. **(B)** Scalp amplitude difference map for the P300 time window (490–540 ms). Stars show electrodes that present significant differences between conditions according to a non-parametric cluster-based permutation test.

Because of the need for a large number of trials for calculating the ERPs, we classified participants in two groups: facilitated or hindered by music, independently of the music’s *a priori* emotional intrinsic characteristics. We found that when music hindered task execution, P300 amplitude was attenuated as compared to the facilitated condition (*p* = 0.00041; Figure 7A). The non-parametric cluster-based permutation test (Figure 7B) confirmed this significant difference (*p* < 0.02), and the spatial distribution of the activity over frontal and left electrodes.

## Discussion

In this study, we have tackled the question of whether emotional dispositions can affect cognitive flexibility in a commonly used card sorting task. In addition to the traditional approach based on behavioral results and self-report questionnaires, we have included in-depth first-person descriptions obtained through a micro-phenomenological interview and used the ensuing categories to orient the analysis of the behavioral and electrophysiological data. Among other results that we discuss below, we consistently found that the intrinsic (expected) valence of the emotional stimuli is not determinant in its effect on the person or their task performance: participants could either feel facilitated or hampered by the emotional disposition generated by the same musical stimulus, depending on how they experienced the situation, affecting both their task performance and the underlying neuronal correlates.

## Third-Person Analysis

### Behavioral Results

Regarding our behavioral results, we found a training effect during the experimental session that was evident despite the existence of a formal training period before the task itself. This effect has not been shown in previous research (Barceló, 2003). We also replicated previous findings, which show faster reaction times for repetition trials compared with switch trials (see for example Barceló, 2003).

We did find an emotional effect on the task: music A was associated with overall faster RTs in comparison to B and S conditions. Interestingly, this effect persists in time so that the RTs of the sequence that started with music A (ABAB) were faster when compared to a sequence starting with music B (BABA). An important conclusion from these results is that emotional states induced in experimental conditions can have long-lasting effects, an issue that should be taken into account when designing experiments that assess the effects of emotional induction (in contrast to emotion recognition) on other cognitive or behavioral processes.

Participants’ reported comparable ratings in both the arousal and valence dimensions for the silent condition and music B (medium-high values for valence and arousal). They differed however in terms of the feelings generated by music A, which were characterized by high arousal and low valence. In this sense, these results suggest that the emotional disposition promoted by music A would be facilitating for cognitive flexibility. It is worth noting that it has been proposed that negative emotions allow a better focus on the task (Andrews and Thomson, 2009). Indeed, negative emotions and their association with problematic environments have sometimes been shown to facilitate precautions and controlled responses (e.g., Bless et al., 1996 in Andrews and Thomson, 2009).

In line with our results, Rowe et al. (2007) hypothesized that positive emotional states relax inhibitory control, resulting in a widened scope of attention. However, because music B promoted a state which was similar to the silence condition, our understanding of the effects of positive emotional states on cognitive flexibility remains limited. Previous studies have shown that positive affect induced by emotional images enhanced task-switching abilities on a set-shifting task, whereas negative affect did not (Dreisbach and Goschke, 2004). This improved switching ability came at the cost of increased distractibility, which is believed to result from poorer task maintenance in working memory during positive affect (Dreisbach, 2006).

Finally, in relation to the duration of the emotional effect, some authors have argued that in order to induce an emotional mood, the stimulus must have a minimum duration of approximately 7 min, and that the effect should last from 5 to 40 min (Västfjäll, 2002) to 3 h or more, in some cases (Oatley et al., 2006). In agreement with this, we induced an emotional state in our participants that was highly dependent on the first emotional condition, and that apparently interfered with posterior mood induction. As stated above, this information is relevant for future research as the duration of the emotional stimuli and the emotional induction could be decisive in terms of experimental design and, in particular, in terms of the number of sessions of the study.

### Electrophysiological Results

As expected, the shift signal evoked a P300 between 490–540 ms in all conditions. An increase in P300 amplitude is associated with the change of a mental set (Barceló, 2003; Periáñez and Barceló, 2009). In our study, P300 was larger in the silence condition compared to both emotional conditions, but no significant differences were found between emotional conditions. As in Barceló (2003) study, the P300 presented a frontal distribution for rule change trials, which then disappeared for rule repetition trials. Several authors have interpreted the P300 as a neural signature of the cognitive control mechanisms required for mental set reconfiguration during perspective changes (Kieffaber and Hetrick, 2005; Nicholson et al., 2005; Kopp et al., 2006). In the context-update hypothesis (Donchin, 1981; Donchin and Coles, 1988 in San Martín, 2012) the P300 is an index of brain activity underlying the revision of a mental model of the current task. This revision is induced by a stimulus that updates the model, being the amplitude of the P300 proportional to the amount of cognitive resources used during the revision of the model. Since music produces a decrease in the amplitude of the P300, it is possible that cognitive resources are used partly by the task and partly by the musical stimulus, regardless of its intrinsic emotional characteristic. Another way to explain these results is that music produces an increase in workload, thus producing a decrease in the P300 component, as reported by Hasegawa et al. (2004).

## Combined First and Third-Person Results

Self-report questionnaires are commonly used to assess participants’ subjective experience in experimental paradigms. However, and despite the wealth of information that they can provide under well-controlled conditions, they remain limited when it comes to obtaining a more nuanced view of the experience, one that is less constrained by the experimenters’ expectations or previous knowledge (Colombetti and Thompson, 2008; Colombetti, 2013). Consequently, the interpretation of the subjective experience is difficult and does not allow to explain, comprehend and interpret the observed behavioral and physiological information that accompanies the experience.

To address this limitation, we used the micro-phenomenological interview to guide participants in their description of this particular experimental situation. As described in the methods section, this approach has been used in a wide range of contexts that require a more in-depth description of a specific experience, while allowing for an open exploration of the participant’s phenomenology (Petitmengin et al., 2007; Valenzuela-Moguillansky, 2013; Ataria, 2015; Depraz and Archives, 2017; Petitmengin, 2017; Vásquez-Rosati, 2017; Przyrembel and Singer, 2018). The analysis of the interview revealed that the different musical stimuli could be perceived as an external object or could induce internal changes in participants. This result supports the proposal of Gabrielsson (2002) in which “perceive” or “feel” an emotion are different processes in response to music. In the current study, we found a positive correspondence between presented and perceived emotions. Music A was perceived as carrying a negative emotion and music B as carrying a positive one. However, when the music was felt, we found positive and negative relationships for both emotional stimuli: both music A and music B produced comfortable and uncomfortable sensations.

Interestingly, independently of the emotional valence of the stimulus, participants could feel hindered or facilitated by the music. This information allowed us to clustered participants according to their experience and then re-analyze behavioral and electrophysiological data. We found significant differences between facilitated and hindered conditions only when music B was played. When the music hindered performance according to the participant’s description, it promoted distractibility and RTs were slower than when the music accompanied the execution of the task by promoting concentration. According to the descriptions of subjective experience, the attentional focus of participants while doing the task with music B could be either on the music or on the task, something that is coincident with the behavioral findings. On the contrary, differences in music A were not so clear. Here, the generic structure of experience showed three possible attentional focuses (music, task/music, and task), which could explain the similitude in the RTs of both conditions.

Brain activity was also grouped according to phenomenological information. Given the low number of trials, however, it was necessary to group all those participants who felt facilitated or hindered in the task, independently of the emotional valence of the musical stimulus. These results showed that when the music hindered the task, the evoked potential P300 decreased its amplitude. As previously reported, the amplitude of P300 is associated with cognitive resources used in task updating (Donchin, 1981; Donchin and Coles, 1988) and workload (Hasegawa et al., 2004). Therefore, the decreased amplitude of the P300 may reflect fewer cognitive resources, associated with the feeling of being hampered in performing the task with music. This may also be accompanied by an increase in workload, given the participants’ constant struggle to bring attention back to the task.

In general, our results reveal individual differences in the relationship between emotional states and cognitive functions. Reaction times and cortical activity results show that not all individuals reacted equally to a stimulus or emotional state when executing a task. Emotional experience is unique to each individual, and the way music alters our mood is in most cases predictable. However, this does not hold when are faced with changing demands in our environment. Faced with situations of change of perspective, the emotional expressiveness of music is not necessarily in line with the emotional disposition required to accomplish a goal and can, therefore, affect it substantially.

The first- and third-person information dialogue in the study of emotional states is a relatively new field that needs to be explored with subtlety. As shown in this study, each emotional experience is unique and so are its electrophysiological correlates. Both types of information require methodologies that allow us to reveal the underlying generic structure, and thus be able to achieve a higher explanatory level of the relationship between psychological and physiological processes.

## Ethics Statement

This study was carried out in accordance with the recommendations of the Ethics Committee of the School of Psychology, from the Pontificia Universidad Católica de Chile. The protocol was reviewed and approved by this Committee. All participants read and signed an informed consent form prior to their participation, in accordance with the Declaration of Helsinki.

## Author Contributions

AV-R contributed to the experimental design, performed the experiments and the interviews, analyzed the behavioral and interview data, contributed to the interpretation of results, and wrote and edited the manuscript. RM-S performed the EEG analysis, and contributed to the writing of the manuscript and critical review of the results. VL contributed to the experimental design, and the critical review and interpretation of the results. DC contributed to the experimental design, and interpretation of the results, and wrote, edited, and critically reviewed the manuscript.

## Conflict of Interest Statement

The authors declare that the research was conducted in the absence of any commercial or financial relationships that could be construed as a potential conflict of interest.
